# Outcomes of an Asynchronous Care Model for Chronic Conditions in a Diverse Population: 12-Month Retrospective Chart Review Study

**DOI:** 10.2196/53835

**Published:** 2024-03-13

**Authors:** Michael Hofner, Patrick Hurnaus, Dan DiStefano, Shaji Philip, Sarah Kim, Julie Shaw, Avantika Chander Waring

**Affiliations:** 1 9amHealth Encinitas, CA United States; 2 Washington Permanente Medical Group Seattle, WA United States; 3 Zuckerberg San Francisco General Hospital, Division of Endocrinology, Diabetes and Metabolism University of California, San Francisco San Francisco, CA United States; 4 The Ottawa Hospital and EORLA University of Ottawa Ottowa, ON Canada

**Keywords:** asynchronous, blood pressure, cardiology, chronic disease, cohort, diabetes mellitus therapy, diabetes, diabetics, eHealth, e-health, HbA_1c_, health disparities, heart, hemoglobin A1c, hypertension therapy, hypertension, hypertensive, remote care, retrospective, telehealth, telemedicine, virtual care

## Abstract

**Background:**

Diabetes and hypertension are some of the most prevalent and costly chronic conditions in the United States. However, outcomes continue to lag behind targets, creating further risk of long-term complications, morbidity, and mortality for people living with these conditions. Furthermore, racial and ethnic disparities in glycemic and hypertension control persist. Flexible telehealth programs leveraging asynchronous care allow for increased provider access and more convenient follow-up, ultimately improving critical health outcomes across demographic groups.

**Objective:**

We aim to evaluate the 12-month clinical outcomes of participants in the 9amHealth web-based clinic for diabetes and hypertension. We hypothesized that participation in the 9amHealth program would be associated with significant improvements in glycemic and blood pressure (BP) control across a diverse group of individuals.

**Methods:**

We enrolled 95 patients in a completely web-based care clinic for diabetes and hypertension who received nutrition counseling, health coaching, and asynchronous physician consultations for medication prescribing. Patients received standard or cellular-connected glucose meters and BP cuffs in order to share data. Laboratory tests were completed either with at-home phlebotomy draws or a self-administered test kit. Patients’ first and last hemoglobin A_1c_ (HbA_1c_) and BP results over the 12-month period were compared, and analyses were repeated across race and ethnicity groups.

**Results:**

Among all 95 patients, the average HbA_1c_ decreased by –1.0 (from 8.2% to 7.2%; *P*<.001) over 12 months of program participation. In those with a baseline HbA_1c_ >8%, the average HbA_1c_ decreased by –2.1 (from 10.2% to 8.1%; *P*<.001), and in those with a baseline HbA_1c_ >9%, the average HbA_1c_ decreased by –2.8 (from 11% to 8.2%; *P*<.001). Among participants who identified as a race or ethnicity other than White, the HbA_1c_ decreased by –1.2 (from 8.6% to 7.4%, *P*=.001). Further examination of subgroups confirmed HbA_1c_ lowering within each race or ethnicity group. In the overall population, the average systolic BP decreased by 17.7 mm Hg (*P*=.006) and the average diastolic BP decreased by 14.3 mm Hg (*P*=.002). Among participants self-identifying as a race or ethnicity other than White, the results similarly showed a decrease in BP (average reduction in systolic BP of 10 mm Hg and in diastolic BP of 9 mm Hg).

**Conclusions:**

A fully web-based model leveraging all-asynchronous physician review and prescribing, combined with synchronous and asynchronous coaching and nutrition support, was associated with clinically meaningful improvement in HbA_1c_ and BP control over a 12-month period among a diverse group of individuals. Further studies should prospectively evaluate the effectiveness of such models among larger populations, assess the longer-term sustainability of these outcomes, and explore financial models to make these types of programs broadly accessible.

## Introduction

Diabetes and hypertension collectively represent some of the most prevalent chronic conditions in the United States, affecting 11% and 45% of adults, respectively [1,2]. Despite the high prevalence of these conditions, improvements in care have lagged. For example, despite increased health care spending on people with diabetes and higher spending on diabetes medications [3], glycemic control has decreased over the past decade [4]. Similarly, rates of hypertension control have declined, with less than 50% of adults with hypertension meeting target blood pressure (BP) in 2020 [5].

When looking at outcomes across racial and ethnic groups, wider gaps in care are realized. The data show a higher incidence of diabetes-related complications in Black and Hispanic populations [6], in addition to racial disparities in glycemic control [7]. Hypertension, which disproportionately affects racial and ethnic minority individuals, is also less often controlled in Black American and Mexican American populations [8].

The causes of these suboptimal outcomes are multifactorial and include geographic and financial barriers to accessing care and broader systemic inequities. Transportation infrastructure and a limited number of providers pose challenges for patients living in rural areas [9]. Affordability is another significant barrier for patients. The data show an increase in national spending on diabetes medications over the past decade, with patients reporting cost-related underuse of critical diabetes medications [10].

Furthermore, over 40% of working-age adults are underinsured (uninsured, gaps in insurance, and inadequate coverage to ensure access to care) and potentially without access to consistent medical care for chronic conditions [11].

Telehealth has become an increasingly common method of care delivery that seeks to address many of these barriers [12-14]. However, the effectiveness of telehealth for chronic conditions remains unconfirmed, and the various telehealth solutions studied are heterogeneous, with some providing remote coaching only and others providing synchronous video visits with a prescribing provider [15-17]. Additional concerns over the effectiveness and value of telehealth include the potential widening of the digital divide and the worsening of health equity gaps [18,19].

This study evaluates the 12-month outcomes of a web-based clinic that is designed to overcome many of these barriers. The web-based clinic under study leverages an asynchronous physician consult model, where orders can be placed after chart and data review, plus relevant information provided by the patient. Asynchronous models reduce costs and increase efficiency and access due to the flexibility of prescriber availability. We hypothesized that participation in the 9amHealth web-based clinic, which combines telehealth coaching, remote monitoring, and asynchronous physician consultation for medication prescribing, would be associated with improvements in both glycemic and BP control over a 1-year period among a diverse group of individuals.

## Methods

### Ethical Considerations

All patients included in this cohort self-enrolled in the 9amHealth program, provided express consent to medical care by telemedicine, and agreed to our terms and conditions, which include authorization to conduct additional research using health care data obtained as part of the program. Ethics review board assessment was not sought as this study is a secondary analysis of previously collected deidentified data, considered secondary research for which consent is not required per federal regulation code 46.104 [20].

### Study Design

This was a nonrandomized, retrospective observational cohort study evaluating the clinical outcomes among members with diabetes and hypertension who were enrolled in the 9amHealth web-based clinic program for 12 months. For inclusion in this analysis, we identified charts from members who enrolled in the 9amHealth program between 2020 and 2022, who remained with the program for at least 12 consecutive months, and who had at least 2 verified hemoglobin A_1c_ (HbA_1c_) laboratory test results recorded.

### Program Description

The 9amHealth program is a web-based clinic for people living with type 2 diabetes, prediabetes, hypertension, hyperlipidemia, and obesity. Participants learn about the program through web-based advertisements, social media groups, and community referrals. Individuals at risk for chronic conditions sign up for an initial screening, and those with new or existing diagnoses pay a monthly subscription fee to enroll in a chronic condition management program. The program’s base fee (US $25 per month at the time of the study) includes unlimited synchronous and asynchronous care from registered dietitians and diabetes educators and unlimited asynchronous care from physicians. At-home laboratory test services and generic medications incur additional fees, with a fee range between US $25 and US $55 per month. Upon enrollment, members provide consent to be treated by telehealth. Members start the program with a web-based medical questionnaire that collects medical history; medications; allergies; and demographic information on insurance status, race, ethnicity, and gender identity. Diagnoses of type 2 diabetes are either self-reported by the patient and confirmed by HbA_1c_ laboratory test results ≥6.5% or determined based on HbA_1c_ laboratory test results ≥6.5% alone. Diagnoses of hypertension are self-reported by the patient or identified by screening BP readings done through the program.

BP cuffs (McKesson, Smart Meter) and glucose meters (Ascencia, Smart Meter) are provided to members based on their condition and the clinical need for monitoring, and continuous glucose monitors are ordered for individuals who meet their health plan’s criteria for these devices. Members are also invited to share data through the program’s app from their personal devices.

### Laboratory Measures

Laboratory tests are ordered on a protocol-driven cadence specified by the 9amHealth clinical algorithm, which aligns with standards of care recommendations from the American Diabetes Association and includes HbA_1c_, a comprehensive metabolic panel, a lipid panel, and a urine microalbumin to creatinine ratio test [21]. In brief, the protocol recommends an HbA_1c_ test every 3-6 months, depending on level of control and medication changes; a comprehensive metabolic panel and urine microalbumin to creatinine ratio tests are repeated annually; and lipid panels are repeated every 2 years unless abnormal results or medication changes necessitate interim testing.

Laboratory tests are collected by an at-home phlebotomy partner, and specimens are processed and analyzed at 1 of the 3 Clinical Laboratory Improvement Amendments of 1988 (CLIA)–certified, College of American Pathologists–accredited laboratories (Quest, Labcorp, or Bioreference). In regions where a phlebotomist cannot be deployed to the home, members are offered an at-home test kit (Molecular Testing Labs dried blood spot, Tasso device) that can measure creatinine, HbA_1c_, and lipid panel, or they can travel to an in-person patient service center. Members can also share laboratory test results ordered by other providers directly into the 9amHealth patient management system.

### BP Readings

BP readings are either self-reported by the member to the care team; through member upload to the app; or, in the case of cellular-connected BP cuffs, automatically uploaded through the device company’s web-based portal.

### Clinical Care

Diabetes education, coaching, and nutrition counseling are provided by Registered Dietitians and Certified Diabetes Care and Education Specialists through a combination of scheduled and unscheduled telephone visits, secure messaging, and SMS text messages. Topics are addressed according to the *Association of Diabetes Care and Education Specialists ADCES7 Self-Care Behavior Guidelines* [22]. No calorie restriction or specific macronutrient counting is required, and recommendations are customized to meet the preferences, lifestyle, and cultural requirements of the member.

After an asynchronous review of the web-based questionnaire; available glucose, BP, and weight data; and any additional clinical information gathered by the registered dieticians and coaches, medications are prescribed by physicians trained on the 9amHealth clinical algorithms. These algorithms are written by endocrinologists and primary care physicians and align with the American Diabetes Association’s guidelines [23] and community standard practice. Algorithms include recommendations (within parameters) for medication management of hyperglycemia, hypertension, hyperlipidemia, and obesity and for addressing abnormal laboratory test results. Medication recommendations are tailored to the member based on other health conditions, side effect profiles, insurance coverage, and acceptability of copays and cost-shares. Within the diabetes algorithm, glucose patterns are identified, and dose escalation or de-escalation of medications or an additional medication is suggested. Similarly, within the hypertension algorithms, an average of 3 BP readings obtained on separate dates is evaluated, and antihypertensive doses are escalated, de-escalated, or an additional medication is suggested. All algorithm suggestions are reviewed by registered dietitians and diabetes educators with the patient and then reviewed asynchronously by the physician in the context of chart review and consultation, and prescription changes are submitted if deemed clinically appropriate. Medications prescribed include metformin, sodium-glucose transport protein 2 (SGLT2) inhibitors, glucagon-like peptide 1 (GLP1) receptor agonists, sulfonylureas, pioglitazone, and long-acting, intermediate-acting, and rapid-acting insulins for glucose control. Generic statins, ace inhibitors, angiotensin receptor blockers, amlodipine, and hydrochlorothiazide are prescribed for the management of hypertension and cardiovascular risk.

### Statistical Methods

Demographic information is reported as the mean (SD) or n (%). The first and last available HbA_1c_ results were compared among all included members, as well as in the subgroups with a baseline HbA_1c_ >8% (poor control group) and a baseline HbA_1c_ >9% (uncontrolled hyperglycemia group) using paired 2-tailed *t* tests. The first and last available BP readings were compared among participants in the cohort with baseline BP ≥140/90 who had at least 2 BP readings, measured at least 1 month apart, and uploaded to the patient management system, which also used a paired 2-tailed *t* test.

## Results

### Participant Demographics

Table 1 describes the baseline and follow-up characteristics of the cohort, subgrouped by self-reported race or ethnicity. The average age of the overall population was 48 years, with 64% (61/95) of participants identifying as men and 34% (32/95) identifying as women. Nearly half of the population self-identified as a race or ethnicity other than White.

**Table 1:** Baseline and follow up characteristics of participants.

Characteristic		Overall population (N=95)	Self-identify as White (n=52)	Self-identify as race or ethnicity other than White (n=39)
Age (years), n (%)		48 (9)	49 (9)	46.5 (10)
**Sex assigned at birth, n (%)**				
	Female	32 (34)	14 (27)	18 (46)
	Male	61 (64)	37 (71)	21 (54)
	Declined	2 (2)	1 (2)	0 (0)
**Race or ethnicity, n (%)**				
	Asian	10 (11)	N/A^a^	N/A
	American Indian or Alaska Native	1 (1)	N/A	N/A
	Black or African American	13 (14)	N/A	N/A
	Latinx	15 (16)	N/A	N/A
	White	52 (55)	N/A	N/A
	Other or unknown	4 (4)	N/A	N/A
Average number of days with the program, mean (SD)		488.5 (75.0)	N/A	N/A
Average baseline HbA_1c_^b^ (%), mean (SD)		8.2 (2.2)	7.8 (2.2)	8.6 (2.1)
Average last HbA_1c_ (%), mean (SD)		7.2 (1.9)	7.1 (2.0)	7.4 (1.9)
**Average baseline BP^c^ (mm Hg), mean (SD)**				
	Systolic	158.7 (16.9)	N/A	N/A
	Diastolic	97.5 (4.5)	N/A	N/A
**Average last BP (mm Hg), mean (SD)**				
	Systolic	141.0 (26.2)	N/A	N/A
	Diastolic	83.3 (12.6)	N/A	N/A
**Number of participants who were prescribed each medication by 9amHealth, n (%)**				
	Amlodipine	9 (10)	N/A	N/A
	Atorvastatin	17 (18)	N/A	N/A
	Glimepiride	4 (4)	N/A	N/A
	Glipizide	8 (8)	N/A	N/A
	Hydrochlorothiazide	7 (7)	N/A	N/A
	Lisinopril	10 (11)	N/A	N/A
	Losartan	9 (10)	N/A	N/A
	Omega-3-acid ethyl esters	3 (3)	N/A	N/A
	Metformin	32 (34)	N/A	N/A
	Pioglitazone	19 (20)	N/A	N/A
	Rosuvastatin	3 (3)	N/A	N/A
	Simvastatin	1 (1)	N/A	N/A
	Dulaglutide	1 (1)	N/A	N/A

^a^N/A: not applicable.

^b^HbA_1c_: hemoglobin A_1c_.

^c^BP: blood pressure.

### HbA_1c_ Results

**Figure 1:**
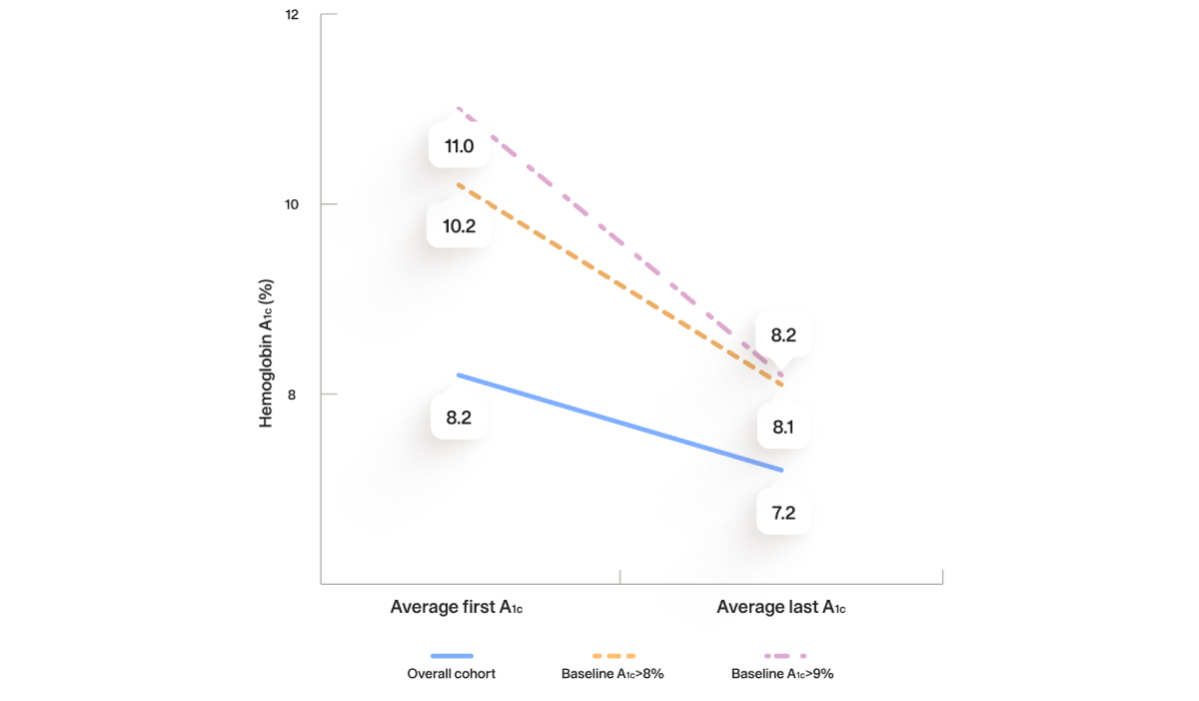
Change in hemoglobin A1c (HbA_1c_) over the study period.

Figure 1 demonstrates the change in HbA_1c_ in all participants and in the baseline HbA_1c_ >8% and >9% cohorts. Among all 95 participants, the average HbA_1c_ decreased from 8.2% to 7.2% (–1.0; *P*<.001), with an average of 314 days between the first and last results. Among participants with a baseline HbA_1c_ >8%, the average HbA_1c_ decreased from 10.2% to 8.1% (n=46; –2.1; *P*<.001). Among those with a baseline HbA_1c_ >9%, the average HbA_1c_ decreased from 11% to 8.2% (n=32; –2.8; *P*<.001).

The results were consistent among members identifying as a race or ethnicity other than White. The average HbA_1c_ among participants who identified as a race or ethnicity other than White decreased from 8.6% to 7.4% (n=39; –1.2; *P*=.001). Further examination of subgroups confirms HbA_1c_ lowering within each race or ethnicity group, however, in small numbers. Among Asian participants, the average HbA_1c_ decreased from 8.8% to 6.9% (n=10; –1.9; *P*=.004); among Black or African American participants, the average HbA_1c_ decreased from 7.5% to 7.1% (n=13; –0.3; *P*=.46); and among Hispanic or Latinx participants, it decreased from 8.9% to 7.9% (n=15; –1.1; *P*=.07). Of note, the baseline HbA_1c_ in Black participants was the lowest of any group, close to target upon starting the program at 7.5%.

### BP Results

**Figure 2:**
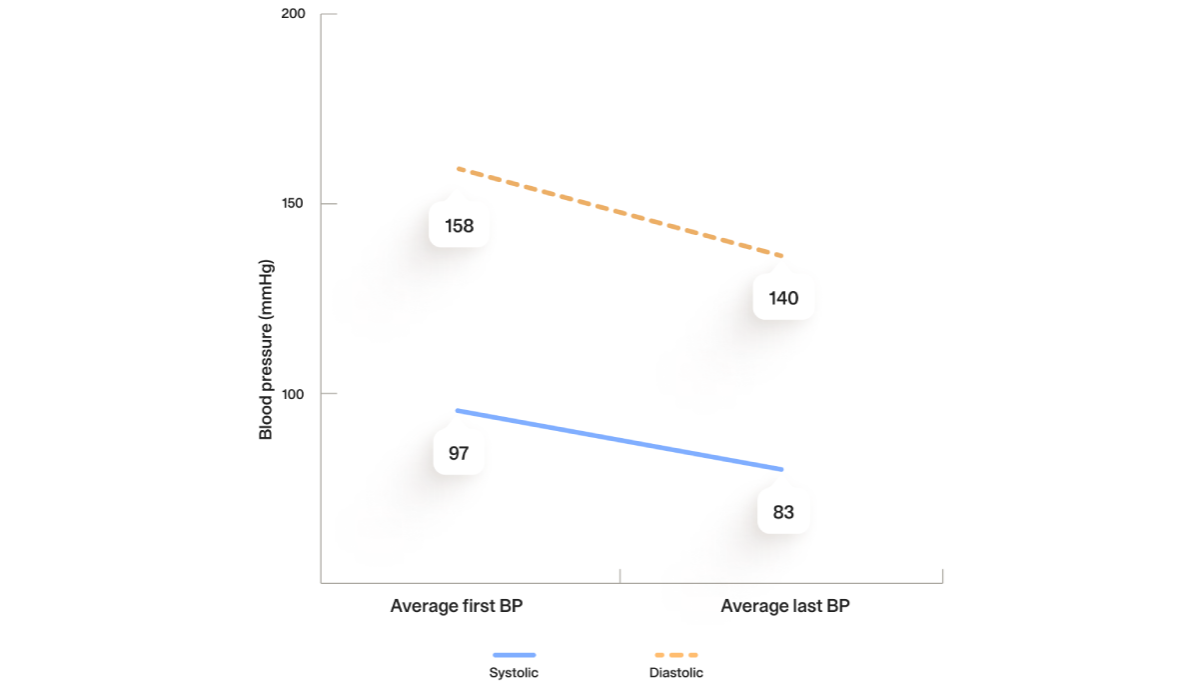
Change in blood pressure (BP) over the study period.

Figure 2 shows the change in BP among all participants in the program for at least 12 months with baseline BP ≥140/90 and available first and last BP readings. The average systolic BP decreased by 17.7 mm Hg (n=12; *P*=.006) and the average diastolic BP decreased by 14.3 mm Hg (n=12; *P*=.002). Among participants self-identifying as a race or ethnicity other than White, the results similarly showed a decrease in BP (average reduction in systolic BP of 10 mm Hg and in diastolic BP of 9 mm Hg), but with a very small number of individuals meeting the criteria for analysis (n=5). Results for BP were not further parsed by race or ethnicity due to the small sample size.

### Clinical Interventions

Participants were prescribed an average of 2.2 active medications for diagnoses of diabetes, hypertension, and hyperlipidemia. Of these, an average of 1.4 medications were new and added through asynchronous 9amHealth physician consultations.

## Discussion

### Principal Findings

Members participating in a fully web-based model leveraging all-asynchronous physician review and prescribing, combined with synchronous and asynchronous coaching and nutrition support, experienced significant and clinically important improvements in HbA_1c_ and BP control over a 12-month period.

### Comparison With Previous Work

It has long been established that intensive glucose control in type 2 diabetes (HbA_1c_ ≤7%) decreases the risk of microvascular complications, including kidney and eye disease and neuropathy, and these benefits are durable over time [24,25]. Hypertension management has also been shown to reduce adverse cardiovascular outcomes, and meta-analysis data from over 400,000 participants demonstrate that a reduction of systolic BP by 10 mm Hg or a reduction of diastolic BP of 5 mm Hg predicts a 25% reduction in coronary heart disease events and a 36% reduction in strokes [26]. Racial and ethnic minority individuals experience a higher burden of chronic condition complications [6], so it is imperative that a web-based program aimed at lowering HbA_1c_ and BP does so effectively for all racial and ethnic groups. Our results support a positive impact on glycemic control and BP across all race and ethnic groups participating in the program.

### Strengths and Limitations

While many digital programs offer web-based or live coaching and nutrition, and select companies provide medication management along with live telehealth encounters, the 9amHealth program is unique in several ways. In addition to the core elements of coaching, diabetes education, and nutrition, it also integrates key components of medical care—laboratory draws and physician consultations—into one digital experience. The program is also unique in its use of asynchronous physician consultation and prescribing. The asynchronous model drives efficiency and scalability and removes barriers that may exist for certain populations when required to participate in synchronous or scheduled visits. It also reduces the impact of the digital divide since SMS text messages and messaging-based asynchronous clinical communications can occur on a mobile phone without the need for high-speed internet, which may not be available for some underresourced and rural populations.

This analysis has several strengths. First, the population studied was diverse, including a greater percentage of racial and ethnic minority individuals (Table 1) than the average US population [27] and most study populations of digital health solutions [16,28,29]. Second, the glycemic outcomes analyzed in this study are defined by laboratory-measured HbA_1c_ and not extrapolated from self-monitored blood glucose readings, as has been done in previous studies [28]. Third, our analysis included participants regardless of baseline HbA_1c_ or BP. Therefore, we can demonstrate a positive association across a population with varying levels of glycemic and hypertension control at the time of their enrollment, rather than just among individuals starting the program with highly uncontrolled conditions. Finally, participants were included only if they remained in the program for 12 months, demonstrating that initial glucose or BP lowering in the early, high-engagement weeks was sustained throughout the year.

Several limitations must be considered. First, program participants became aware of the program predominantly through advertisements and self-referral. Therefore, the study cohort may represent a motivated population that is more likely to improve health measures such as HbA_1c_ and BP and to engage successfully in digital health solutions. This may have positively impacted the outcomes, suggesting greater HbA_1c_ and BP reductions. Second, nearly half of our participants lack insurance coverage or were enrolled in a high-deductible health plan and, therefore, could not otherwise easily access or afford traditional care. Thus, our results may not generalize to a broader population of predominantly insured individuals. Third, while the population included in this analysis is more diverse than previous studies of digital health solutions, the sample size for racial and ethnic minority individuals was small. Fourth, the financial burden of a monthly subscription fee, although relatively low-cost, may not be sustainable for many individuals in the long term. Therefore, associated reductions in HbA_1c_ and BP may not be sustainable or may only be sustainable for individuals with financial means to remain with the program. Finally, our analysis does not include a comparison to “usual care” or a control group, so the impact of the intervention in isolation cannot be fully separated from other confounding factors. However, existing data suggests that usual care results in a smaller decrease in HbA_1c_ (from –0.5 to –0.9) [16,30,31] than seen with our intervention, which supports the improvement of outcomes seen with the 9amHealth program beyond that of usual care.

### Future Directions

The 1-year outcomes of this web-based clinic demonstrate that participation in a flexible digital health program leveraging asynchronous care is associated with improved chronic condition outcomes beyond just initial engagement in a diverse group of individuals. Future prospective studies, including a comparison control arm, should examine the effectiveness and longer-term sustainability of glucose and BP lowering through this model and evaluate which elements of care are most strongly associated with improved outcomes. Coverage through existing health plans, employer-sponsored programs, and public health benefits should be explored to ensure long-term, affordable access to these types of programs. Finally, studies of larger populations to allow for appropriate power to determine if outcomes are consistent across race or ethnicity groups and broader age groups will allow for further generalizability of these findings.

## Abbreviations

BP: blood pressure
CLIA: Clinical Laboratory Improvement Amendments of 1988
GLP1: glucagon-like peptide 1
HbA_1c_: hemoglobin A1c
SGLT2: sodium-glucose transport protein 2

## Data Availability

The data sets generated during and/or analyzed during this study are available from the corresponding author on reasonable request.
